# Assessment of intention to use contraceptive methods with spatial distributions and associated factors among women in Ethiopia: evidence from EDHS 2016

**DOI:** 10.1186/s13690-021-00631-2

**Published:** 2021-06-21

**Authors:** Girma Gilano, Samuel Hailegebreal

**Affiliations:** Department of Health Informatics, School of Public Health, College of Medicine and Health Sciences, Arba Minch University, Arba Minch, South West Ethiopia

**Keywords:** Intention, Ethiopia, Contraceptive method use, Spatial analysis, EDH data

## Abstract

**Background:**

Modern contraceptive methods have immense influences on the health of mothers and their children. Using contraceptive methods is seen to control family size and unnecessary pregnancies. Considering different factors like resources and various cultural aspects, assessing the intention to use contraception might bring areas with these problems into the light for intervention.

**Methods:**

We analyzed the cross-sectional survey data from EDHS 2016, which comprised 5651 reproductive-age women. Spatial autocorrelation was checked with global Moran’s statistics, at ±1 for dispersion and clustering. Aselin Local moran’s statistics also indicated types of clusters. Hot spot(Getis-Ord Gi) statistics further used to measure autocorrelation over different spatial locations. The significance level was checked by calculating Z-score and hot and cold spots indicated the variation in intention to use contraceptives per catchments. Interpolation was also applied to see the number of intents to use contraceptive areas other than the sampled using ordinary Kriging spatial interpolation. We used Kulldorff’s SatScan for specific local clustering and the Bernoulli model test was applied to check significance. Individual and community-level factors were examined using multilevel logistic regression. Due to the clustering nature of data where *p*-value< 0.05 signaled associations. The disproportional nature of data was adjusted using sampling weights.

**Result:**

From the total sample of women, the intention to use contraceptive methods was 2366.08(44.11%) and was highly clustered in North and Western Ethiopia. The mean number of children was (4.5 ± 2.90); age at first cohabitation was (16.9 ± 3.99); the ideal number of children was (4.77 ± 2.00). Age and the ideal number of children were negatively associated with the use of contraception. Primary education, number of children, counseling at health facilities, and age at first cohabitation were negatively associated.

**Conclusion:**

We observed various distributions among regions. Educational status and various socio-cultural including working with the religious organization might need serious considerations to increase the intention to use contraceptive methods. Besides the efforts done, policy decisions might need to consider this finding and uphill the intervention against the negatively associated socio-cultural and demographic variables in outplayed areas.

## Background

Irrespective of the type of modern contraceptive method women might intend to use, there were no restrictions forwarded by the World Health Organization (WHO) regarding method choices unless unacceptable health risks outweigh the worth of utilizing [1]. Using modern contraceptive methods has got enormous influences on the health of mothers and children. Using the methods have been instrumental to control family size and unnecessary pregnancies. These lead to a safe life for the mother and child and a safer economy for the family size. A woman may incline toward contraceptive method use even though many factors might make things difficult for her. The intention to use contraceptive methods is associated with some socio-demographic variables in Africa [2]. The socio-demographic factors of the women like age, place of residence, marital status, education, religion, work, frequency of visit to health institution, and awareness of the ovulation cycle have reported affecting intention to use contraception methods [3]. According to the studies in East Africa, contraception use was also correlated to parity and attitude of women [4]. Those socio-demographic factors were common in most African countries to affect the intention to use contraceptives. It became explicit when marital status, wealth category, education level, place of residence, number of children, age, religion, and access to a health facility reported in East, South, and West African countries as influencing factors [4–8]. In Ethiopia, data from EDHS (Ethiopian demographic health survey) 2011 and 2016 indicated implications of contraception use on under-five mortality magnitude [9]. There were some evidences showing that a conceivable variation was observed among regions and city administrations regarding contraceptive method uses from EDHS 2016 [10]. However, another study informed a continuous assessment of contraceptive use and their drawbacks, risks, and increasing societal commitment regarding the expansion of them to reduce the risk of unwanted pregnancy is necessary [11]. Educating women, improving women’s awareness, involving males in counseling, and focusing on postpartum contraceptive initiation were advised frequently in developing countries to increase intention to use contraception [12]. Other factors from studies included: sharing information with husband/partner, counseling for contraceptives, education more than primary, the desired number of children were correlated with intention to use [13]. Women from educated families reported using contraceptive methods most of the time for emergency purposes [14]. In Ethiopia, postpartum contraceptive use is the strategy to increase contraception use. However, the usage is still below par. Reasons like cultural and traditional barriers; low male approval; low antenatal visits; inadequate knowledge; and fertility advice showed more correlation [15]. From the study conducted on married women in the same year 2016, we are aware that the intention to use contraceptive method was 44.1%. During that time, the number of children alive, age at marriage, media exposure, employment status, education (both partners), and information obtained from health facilities had positive associations [16]. Even though the intention to contraceptive methods increased 15 folds in some regions in Ethiopia in the last 20 years, the level of utilization and intention to use remains below par. Attitude, religion, and husband involvement grabbed hefty responsibilities for decrements [12, 17–20]. There is a policy under implementation that states, increasing the use of contraceptives from 42% in 2015 to 55% in 2020 [21]. Vigorous assessment of the realization of this policy with spatially informing studies is articulated crucial. Some studies applied multilevel analysis to identify the undertaking indicated implementation status and discovered some clusters in different regions [10, 22, 23]. Despite this, most studies in the country were dealing with contraceptive prevalence, side effects, and socio-demographic factors. Regarding the distribution of intention to use contraceptive methods and its spatial mapping, there was little information in the country. That means studies used contraceptive methods as objective remained limited to picture out the direction for planning and policy decisions. Thus, the large-scale studies which portrait the contraception intention status might give a piece of representative knowledge for planning and policy decisions. Therefore, the current study used country-represented data from EDHS 2016 to discover the unmet need in contraceptives uses with its predictors and spatial distributions.

## Methods and materials

### Data source and participants

We employed a cross-sectional study design data from Ethiopia Demographic Health Survey (EDHS) 2016. Ethiopia is the country located in Eastern Africa (3o-14oN, 33o – 48°E) and is the 2nd most populous country in Africa. EDHS is the country representative sample survey carried out every 5 years interval started in 2000. Ethiopia has nine regions (Afar, Amhara, Benishangul-Gumuz, Gambela, Harari, Oromia, Somali, Southern Nations, Nationalities, and People Region (SNNPR), and Tigray), and two town administrations (Addis Ababa and Dire-Dawa). We retrieved the data for this study from the EDHS website www.dhsprogram.com after the request to access the data approved and downloading allowed. Then 5651 reproductive age (15-49 years) women who never used contraceptive methods were pooled from the dataset of EDHS 2016.

To access the data, the measure program requests registration and permission. We used the data obtained only for the research purpose. We kept it confidential; we avoided any effort of identifying households or individuals. The EDHS data collection obtained permission from Ethiopian Health Nutrition and Research Institute (EHNRI) Review Board and the National Research Ethics Review Committee (NRERC) at the Ministry of Science and Technology. During the data collection, they collected verbal informed consent from participants and explained the purpose as it was published in the 2016 EDHS report [24].

### Study variables

The dependent variable for this study is the intention to use contraceptive methods. We measured the variable as use later (1 = yes) and unsure about use or not intend (0 = no).

#### Individual level variable

Pregnancy termination, heard family planning on the radio in the last few months, read family planning in newspapers/magazines in the past few months, heard family planning on television in the past few months, ever terminated pregnancy, visited field worker in the last 12 months, went to a health facility in the past 12 months, told family planning at a health facility, working status, age, highest education achieved, wealth index, religion, total children ever born, the ideal number of children, husband education, and age at first cohabitation.

#### Community level variables

Region and type of place of residence.

### Statistical analysis

#### Descriptive statistics

Descriptive statistics was presented using, weighted frequencies, mean (standard deviations), and percentage. To adjust for disproportional distribution of sampled size to strata and regions, sampling weights was applied to estimate all descriptive statistics.

#### Spatial analysis

When the subjects in a study have a linear relationship with the dependent variable, traditional regressions are the possible choice for analysis. In other words, when data are structured, clustered or hierarchal, the assumption of linearity is rarely achieved. In our case, there were clusters differs at individual and community levels (hierarchies). Thus, since the data showed due to the hierarchical nature and inter-cluster variation, we assumed multilevel logistic regression to account for variations. Our analysis takes into account, the within and between community variations. To know community effect, Intra-community Correlation (ICC) was estimated using the community level variance. Then the Likelihood Ratio (LR) test, Median Odds Ratio (MOR), and Proportional Change in Variance (PCV) were examined for the fitness of the model as follow [23, 25, 26].

$\mathrm{ICC}=\frac{{\upsigma^2}_a}{{\upsigma^2}_a+{\upsigma^2}_b}$; where, σ^2^_*a*_ is the community level variance and σ^2^_*b*_ indicates individual level variance. The individual variance (σ^2^_*b*_) equal to π^2^/3 that is the fixed value.

$\mathrm{MOR}=\mathrm{MOR}=e\ 0.95\ast \sqrt{V{a}_{\_1}}$, where, *Va*__1_ is the variance in the empty model.

$\mathrm{PVC}=\frac{Va\_1- Va\_2}{Va\_1}$, where, *Va* _ 1 is variance of the empty model and *Va* _ 2 is neighborhood variance in the subsequent model).

To prepare data for spatial (ArcGis), we cross-tabulated the weighted frequency of dependent variables and cluster numbers to obtain the case to total proportion ratio. Then, we combined results with coordinate data. We cleaned and dropped data with zero Latitudes/longitude coordinates and then applied spatial analyses using ArcGIS 10.7 to evaluate whether the pattern of data was clustered, dispersed, or random across the study area.

#### Moran analysis

The spatial autocorrelation (Global Moran’s I) was used to measure if patterns of intention to use contraceptive methods are dispersed, clustered or random in the study area. Moran’s I output varies between (− 1 to + 1). Values close to − 1 indicated an intention to use contraceptives is dispersed whereas, a value close to + 1 indicated intention to use contraceptives is clustered and distributed randomly.

#### Hot spot analysis (Getis-Ord Gi* statistic)

The calculated Gettis-OrdGi* statistics was used to discern how spatial autocorrelation of intention to use contraceptive methods fluctuates across different locations in Ethiopia. Hotspot analysis provides a Z-score and *p*-value which pinpoints the statistical significance of the clustering of target variable over the study area at different significance levels instantaneously. Statistical output with high GI* indicates a “hotspot”(high intention to use contraceptive methods) whereas low GI* means a “cold spot”(low intention to use contraceptive methods) [27].

#### Spatial interpolation

Ordinary Kriging and empirical Bayesian Kriging were considered in this study for they incorporate spatial autocorrelation and statistically optimize the weight. The ordinary Kriging spatial interpolation method was used for predictions of intention to use contraceptive methods in unobserved areas of the country.

#### SaTscan statistics

SaTScan Version 9.6 software was used for the local cluster detection. A circular window that moves systematically throughout the study area was used to identify a significant SaTScan clustering of intention to use contraceptive methods. The data preparation methods of ArcGIS and SaTscan are similar except using yes and no as they are in SaTscan instead of proportion in ArcGIS. We presented the results of primary and secondary observed clusters using relative risk (RR) and log-likelihood (LL).

#### Multilevel binary logistic regression

During multilevel binary logistic regression analysis, we built four models. The first model was intercept-only. It showed the variation among clusters. The second model included individual-level (fixed effect) variables, and the third model contains community-level (random effect) variables. The fourth model (mixed-effect) included all individual and community-level variables. Each variable was filtered at a *p*-value < 0.25 before data analysis. We maintained the statistical significance at a p-value < 0.05 with an adjusted odds ratio and 95% confidence interval also presented. We compared models using deviance (−2LL); the lower deviance indicated good model fitness.

## Results

### Intention to use contraceptive

A total of 5651 reproductive age (15-49 years) women population extracted from the EDHS 2016 dataset, 2366.08(44.11%) had an intention to use contraceptive methods.

#### Individual-level characteristics

The mean number of children ever born to the women in the survey was 4.50 ± 2.90; the mean age at first cohabitation was 16.90 ± 3.99, and the mean ideal number of children reported was 4.77 ± 2.00. Two-third (68.00%) of women were illiterate (no education), and 1/4th learned at least primary school. The information sources experienced by women were radio (18.34%), TV (11.09%), and newspaper/magazine (2.36%). Only 26.14% of participants had visited health facilities, and 43.66% had met with community health workers in the last 12 months. Indeed only 12.32% of them have ever terminated a pregnancy. We also described women by other socio-demographic variables and found Muslims and Orthodox Christians as larger religions. During the survey, only 27.68% of women have a job. Economically 45.61% of women were poor, while 34.48% of them were rich. The age groups with higher participants were 25–29 (19.98%), 30–34 (19.49%), and 35–39 (17.00%). More than half (51.61%) of the women were living with illiterate husbands.

#### Community level characteristics

Most participants were from Oromo (43.78%), Amhara (19.51%), and 19.09% SNNP regions. Greater than three-fourth (88.22%) of the women were from rural-based places of residences (Table 1).

**Table 1:** The descriptive characteristics of the study participants extracted from EDHS-2016 for reproductive women (15-49 years)

Variables	Weighted frequency (%)
**Highest educational level**	
No education	3717.96 (68.12)
Primary education	1370.29 (25.10)
Secondary education	220.00 (4.03)
Higher education	150.04 (2.75)
**Heard FP on the radio**	
No	4380.84 (81.66)
Yes	983.67 (18.34)
**Heard FP on TV**	
No	4769.61 (88.91)
Yes	594.92 (11.09)
Read FP on newspaper	
No	5237.77 (97.64)
Yes	126.76 (2.36)
**Visited by field workers**	
No	3962.44 (73.86)
Yes	1402.10 (26.14)
**The visited Health facility**	
No	3022.46 (56.34)
Yes	2342.10 (43.66)
Ever had birth terminated	
No	4744.78 (87.68)
Yes	666.71 (12.32)
**Region**	
Tigray	365.50 (6.70)
Afar	72.36 (1.33)
Amhara	1064.77 (19.51)
Oromia	2389.00 (43.78))
Somali	261.20 (4.78)
Benishangul	69.70 (1.28)
SNNPR	1041.80 (19.09)
Gambela	16.70 (0.31)
Harari	13.90 (0.26)
Addis Ababa	132.30 (2.42)
Dire Dawa	30.20 (0.55)
**Types of residence**	
Urban	643.15 (11.78)
Rural	4815.10 (88.22)
**Religion**	
Orthodox	1916.24 (35.41)
protestant	1114.60 (20.60)
Muslim	2297.38 (42.45)
Others	83.26 (1.54)
**currently working**	
No	3879.54 (72.32)
Yes	1484.00 (27.68)
**Wealth status**	
Poor	2468.02 (45.61)
Middle	1077.75 (19.92)
Rich	1865.72 (34.48)
**Contraceptive intention**	
no	2998.45 (55.89)
yes	2366.08 (44.11)
**Media exposure**	
**No**	3556.40 (66.29)
**Yes**	1808.20 (33.71)
**Husband education**	
No education	2768.40 (51.61)
Primary education	1905.26 (35.52)
Secondary education	412.70 (7.69)
Higher education	278.10 (5.18)
**Age**	
15–19	312.90 (5.73)
20–24	762.10 (13.96)
25–29	1090.30 (19.98)
30–34	1063.50 (19.49)
35–39	927.76 (17.00)
40–44	665.82 (12.20)
45–49	635.80 (11.65)

### Spatial analysis

#### Moran analysis

**Fig. 1:**
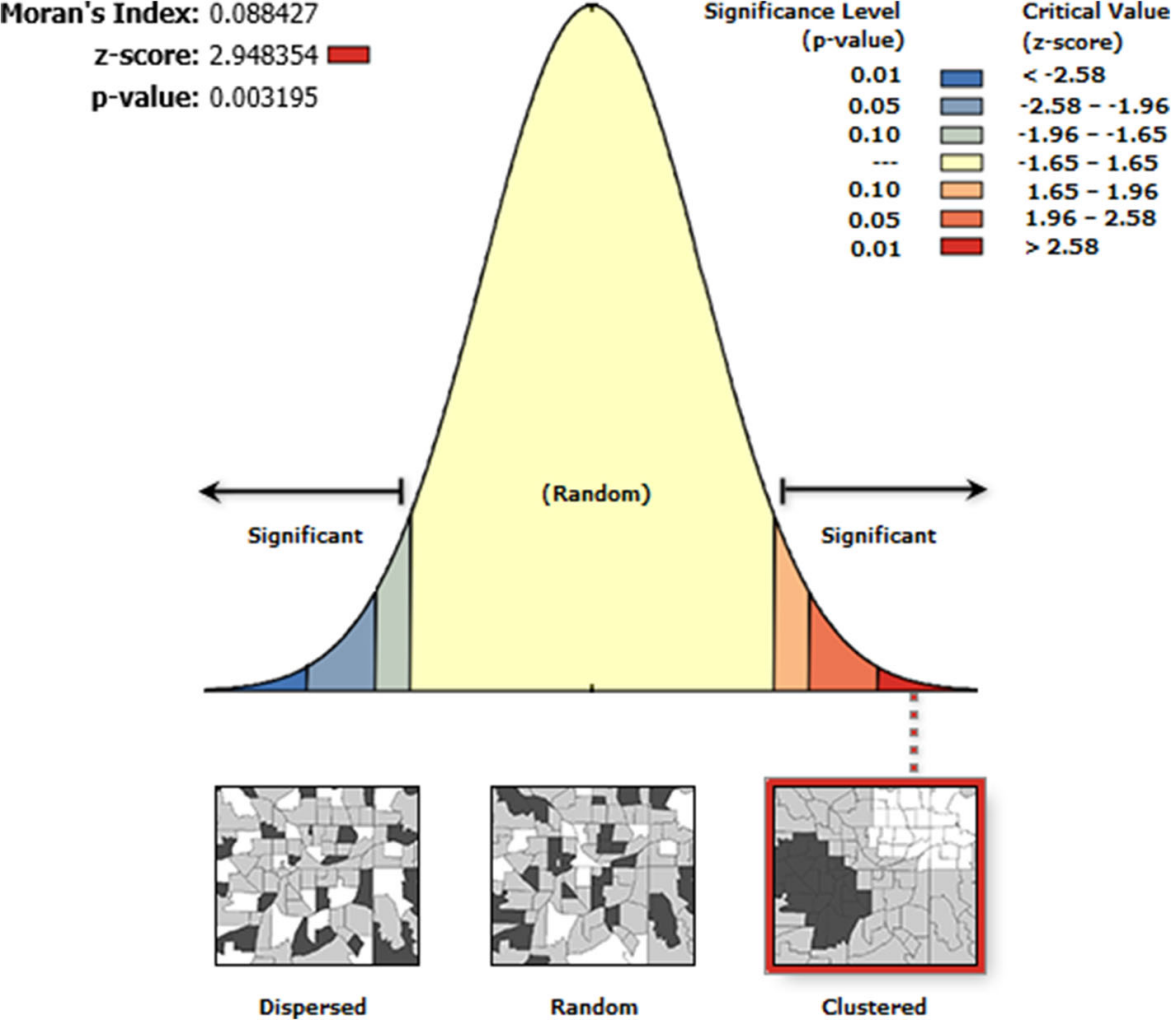
spatial autocorrelation intention to use contraceptive method in Ethiopia, EDHS 2016

From spatial autocorrelation, we found that the distribution of intention to use contraceptive methods in Ethiopia was non-random. The global Moran’s I test is 0.88(*p* < 0.01) show a significant association which is an indication of the presence of a non-randomly distributed intention to use contraceptive methods (Fig. 1). By definition, when global Moran’s I is significant and greater than zero, then the distribution was taken as clustered, while Moran’s test less than zero considered as dispersed. In our analysis, global Moran’s I test 0.88(*p* < 0.01) is evidence of clustered distribution, and the null hypothesis of random distribution was not accepted.

#### Hotspot and cold spot analysis

**Fig. 2:**
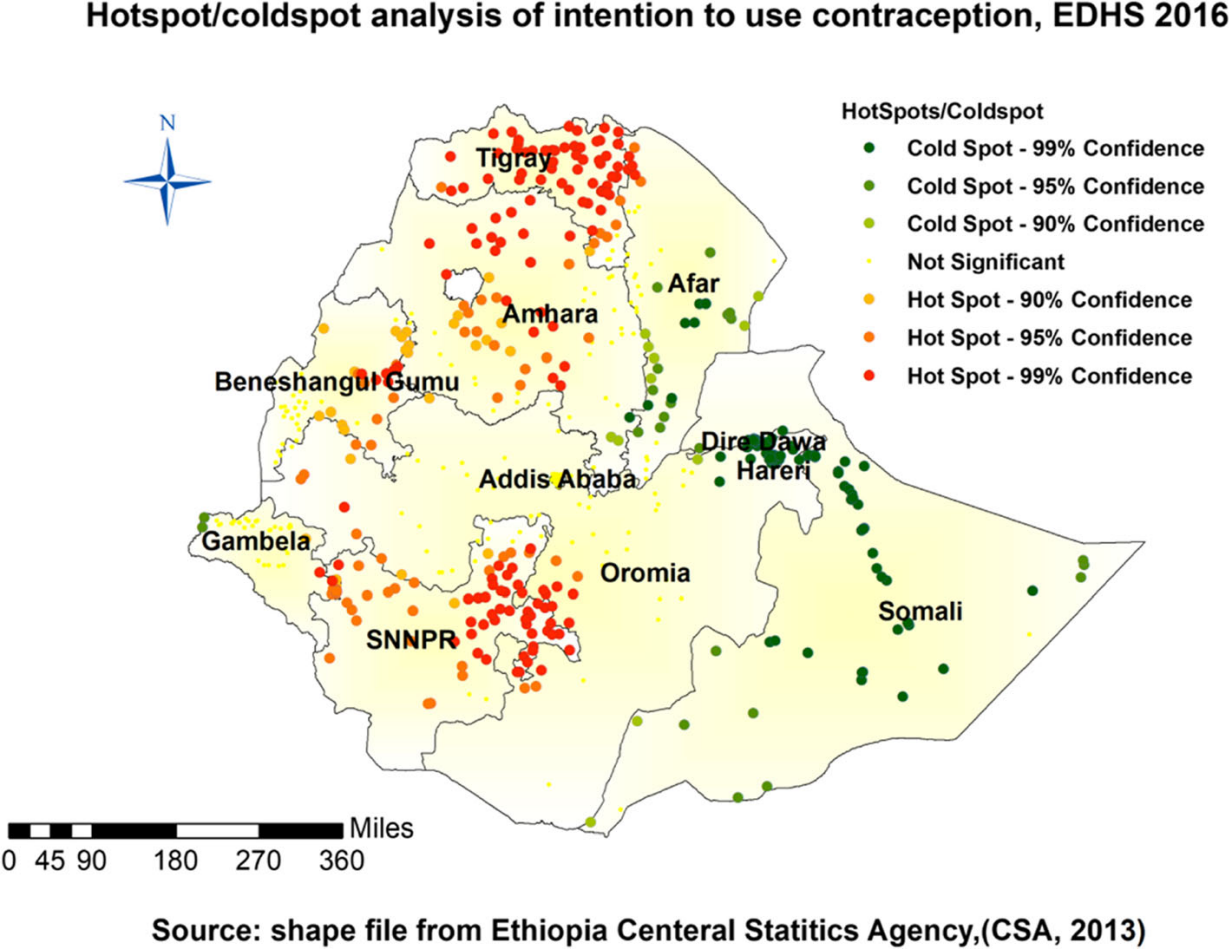
Hot spot and cold spot of intention to use contraceptive methods in Ethiopian, EDHS 2016

The geographical distribution of intention to use contraceptive methods was non-random in the country. Based on the Local Moran’s I, Gettis-OrdGi* statistics analysis, Gambella, Benishangul, Addis Ababa, SNNP, Tigray, Amhara, and Oromia were the regions where the highest prevalence of intention to use contraceptive methods were pragmatic (Fig. 2)

#### Ordinary kriging interpolation

**Fig. 3:**
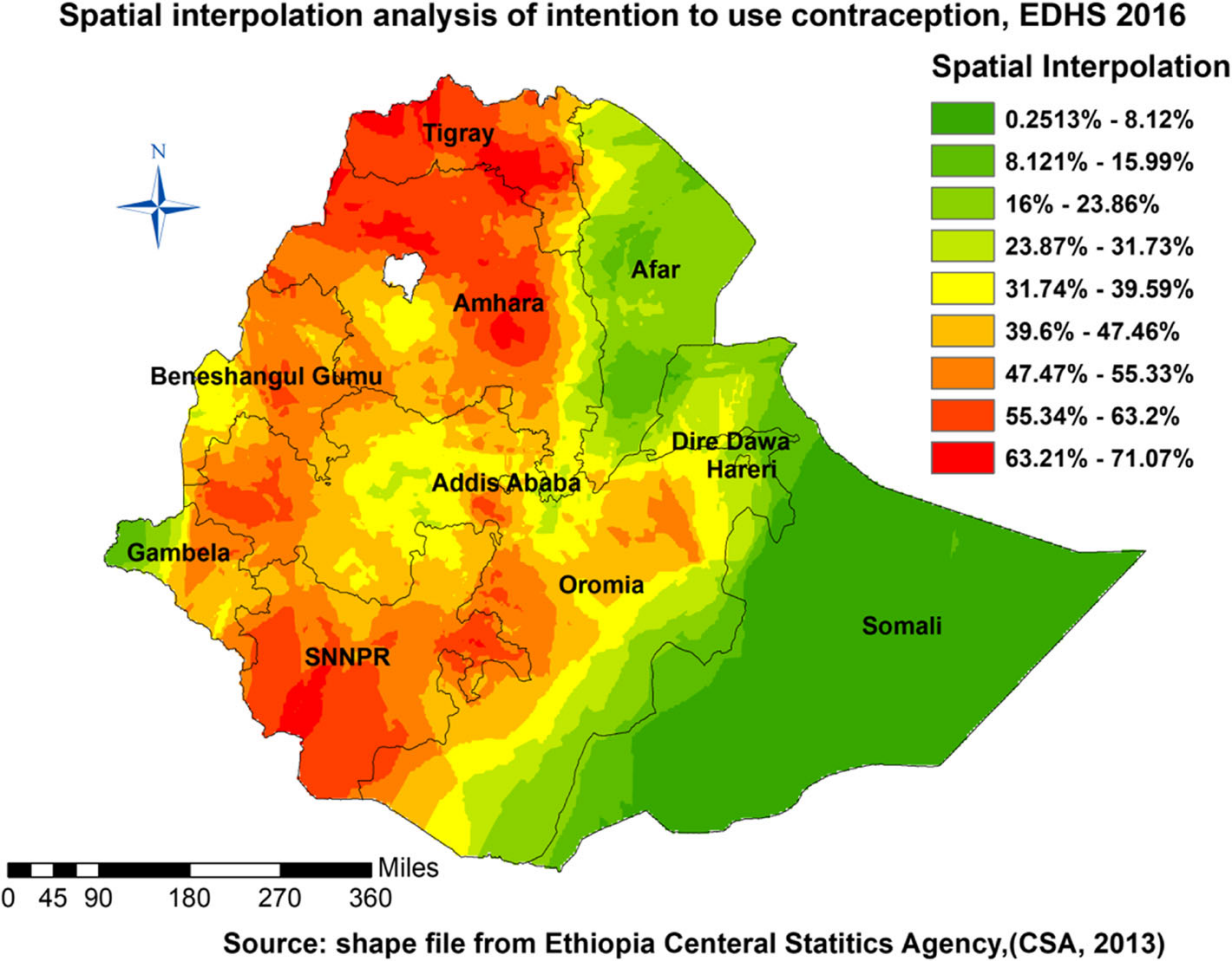
Ordinary Kriging interpolation of intention to use contraceptive in Ethiopia, EDHS 2016

As per the geostatistical scrutiny, the highest prevalence of intention to use contraception was spotted in Amhara, SNNPR, Tigray, and some parts of Oromia, Addis Ababa, Benishangule Gumuz, and Gambella (Fig. 3). Ordinary Kriging interpolation measures the distance from the known point to unknown points/areas to indicate the points in the ranges of the event occurrence. The areas indicated significant (Fig. 3) were the area where the event happening (prediction) was possible..

#### SaTscan statistics

**Fig. 4:**
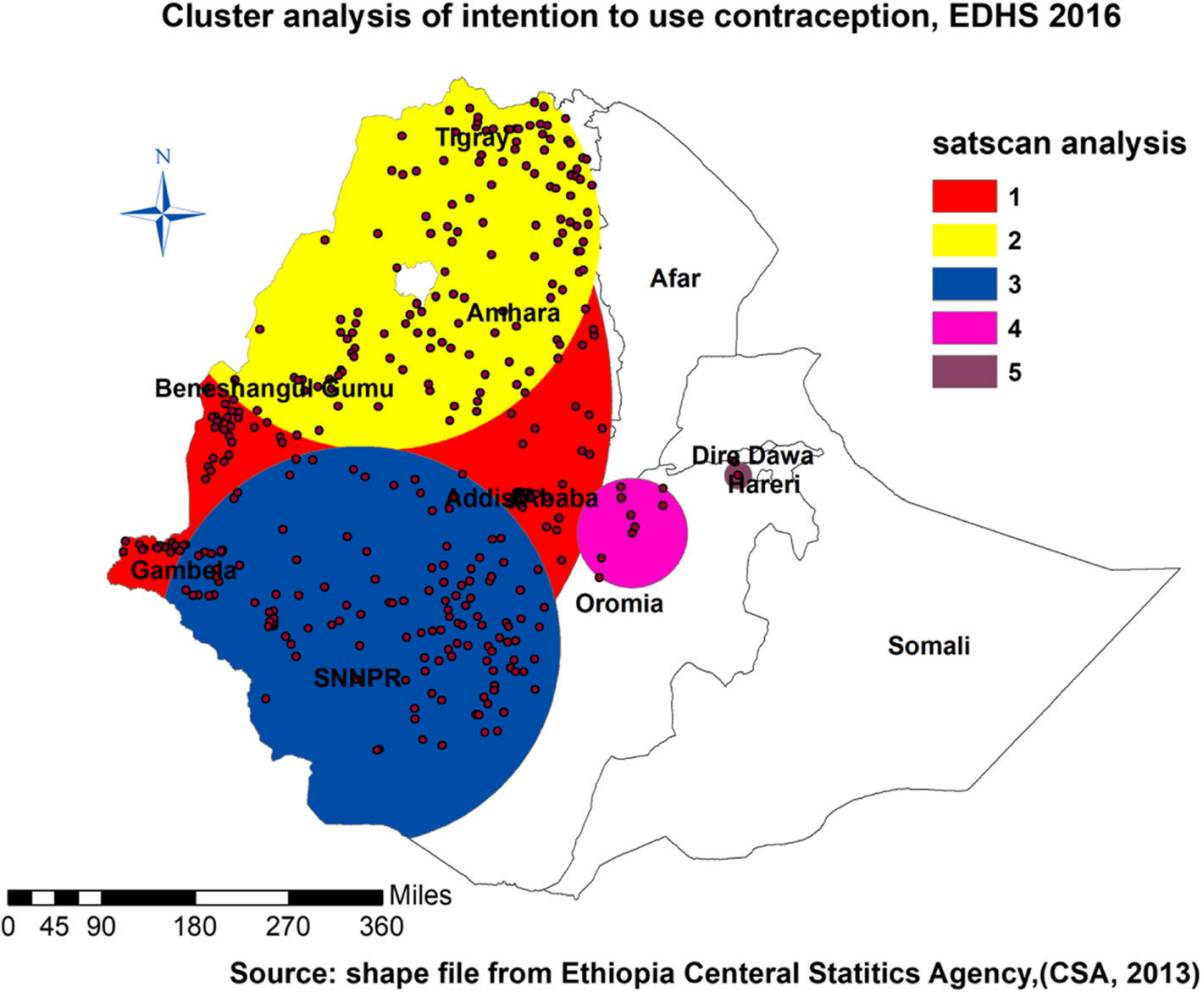
SaTScan scan statistics of intention to use contraceptive method in Ethiopia, EDHS 2016

The data from EDHS 2016 showed a total of three significant local cluster areas. Depending on Table 2 and Fig. 4, the data has one primary/most likely cluster among three significant. A total of 343 locations (Coordinates/radius - 10.298371 N, 34.649187 E) / 588.81 km) with RR of 1.88 and LL of 141.81 were found in the primary cluster. The regions included were Gambella, Benishangul, Addis Ababa, and some parts of SNNPR and Amhara.

**Table 2:** Most likely clusters of intention to use contraceptive methods among women of childbearing age based on the EDHS 2016

Clusters	Observed case	Expected cases	RR	LLR	*p*-value
1	1.31	959.26	1.88	141.80	< 0.001
2	1.56	410.86	1.83	120.44	< 0.001
3	1.35	436.40	1.50	50.70	< 0.001
4	1.60	31.90	1.61	8.50	0.067
5	1.84	8.15	1.85	4.21	0.98

The next cluster (secondary) was located in Tigray and Amhara regions at (12.669915 N, 36.775082 E) with a radius of 335.28 km. And the third significant cluster (secondary) contained the whole SNNPR, some part of Oromia, Gambella, Addis, and Benishangule at (6.934084 N, 36.520510 E) with the radius of 308.29 km, and relative risk of 1.50; (Table 2 and Fig. 4). Individual and community Level factors.

Since we used the data collected on various clusters, we decided to check for the clustering effect among sampling units. High dissimilarities and a larger ICC confirmed variation among clusters. We handled these variations via multilevel logistic regression analyses. To account for the inter-cluster differences (Tables 3 and 4), we built four models. The initial (null) model was the model without predictors (intercept only model) followed by the I-III model buildings for the individual and community level predictors. At individual level, compared to age group 15–19, women in age groups of 25–29,30-34,35-39,40–44, 45–49 had 58.00, 72.00, 90.00, 97.00, and 99.00% had reduced intention to use contraceptive methods with AOR of 0.42(0.30–0.70), 0.28(0.17–0.50), 0.1(0.05–0.18), 0.03(0.02–0.06), and 0.010(0.004–0.02) respectively. Reproductive age women with primary education had a 1.40 likelihood of higher intention to use contraceptives with an AOR of 1.40(1.04–1.80) relative to none educated women. As the number of ever born children increased, the women showed intention for contraceptive use increased with an AOR of 1.10(1–1.20); otherwise, the women with a larger ideal number of children had 11.00% reduced intention to use contraceptive methods with an AOR of 0.89(0.84–0.94). The women who obtained information to use contraceptives at health facilities showed 1.6 times more intention with an AOR of 1.60(1.30–2.10). As age at first cohabitation increased, intention to use contraceptive methods raised 1.04 times with an AOR of 1.04(1.00–1.06).

**Table 3:** Multilevel logistic regression on individual and community-level factors associated with the women (15–49) intention of modern contraceptive methods use

Variables	Model 0	Model I	Model II	Model III
**Age**				
15–19	–	1		1
20–24	–	0.70 (0.45–1.11)		0.71 (0.45–1.13)
25–29	–	0.47 (0.29–0.76)**		0.42 (0.30–0.70)***
30–34	–	0.34 (0.20–0.56)***		0.28 (0.17–0.5)***
35–39	–	0.11 (0.06–0.21)***		0.10 (0.05–0.18)***
40–44	–	0.04 (0.02–0.08)***		0.03 (0.02–0.06)***
45–49	–	0.012 (0.01–0.03)***		0.010 (0.004–0.02)***
**Highest education level achieved**	–			
No education	–	1		1
Primary education	–	1.40 (1.10–1.80)**		1.40 (1.04–1.80)*
Secondary education	–	1.20 (0.80–2.00)		1.30 (0.80–2.10)
Higher education	–	1.30 (0.74–2.40)		1.40 (0.76–2.50)
**Total number of children**	–	1.20 (1.10–1.22)***		1.10 (1–1.20)***
**Told FP at HF**	–			
No	–	1		1
Yes	–	1.70 (1.30–2.10)***		1.6 (1.30–21)***
**Age at cohabitation**	–	1.02 (0.99–1.05)		1.04 (1.00–1.06)*
**Ideal N**^**o**^ **of children**	–	0.90 (0.85–0.95)***		0.89 (0.84–0.94)***
**Husband education**	–			
No education	–	1		1
Primary education	–	1.40 (1.10–1.75)*		1.36 (1.10–1.70)
Secondary education	–	1.10 (0.78–1.60)		1.20 (0.82–1.73)
Higher education	–	0.90 (0.57–1.40)		0.96 (0.60–1.50)
**Religion**	–			
Orthodox	–	1		1
Protestant	–	0.62 (0.44–0.88)**		0.69 (0.48–0.98)
Muslims	–	0.27 (0.20–0.35)***		0.29 (0.22–0.38)
Others	–	0.60 (0.20–1.78)		0.67 (0.23–1.98)
**Wealth status**	–			
Poor	–	1		1
Middle	–	1.40 (1.02–1.90)*		1.40 (1.04–1.90)
Rich	–	1.40 (1.10–1.92)*		1.60 (1.20–2.10)
**Region**	–			
Tigray	–		1	1
Afar	–		0.10 (0.07–0.15)***	0.11 (0.06–0.20)***
Amhara	–		0.77 (0.55–1.10)	1.05 (0.66–1.60)
Oromia	–		0.51 (0.37–0.69)***	0.39 (0.23–0.64)***
Somali	–		0.03 (0.02–0.05)***	0.04 (0.02–0.09)***
Benishangul	–		0.52 (0.36–0.73)***	0.42 (0.25–0.72)**
SNNPR	–		0.72 (0.51–0.99)*	0.79 (0.46–1.36)
Gambela	–		0.34 (0.24–0.0.50)***	0.36 (0.20–0.67)***
Harari	–		0.20 (0.13–0.29)***	0.15 (0.08–0.28)***
Addis Ababa	–		0.30 (0.20–48)***	0.34 (0.20–0.62)***
Dire Dawa	–		0.20 (0.13–0.31)***	0.20 (0.11–0.36)***
**Residence**	–			
Urban	–		1	
Rural	–		0.69 (0.54–0.86)**	0.87 (0.52–1.30)

**NB**: * = *p* < 0.05, ** = p < 0.01, & *** = *p* < 0.001; FP = family planning, south nation nationality people; HF = health facility

**Table 4:** Multilevel logistic regression model comparison and random effect distribution as examined for intention to use contraceptive methods among reproductive age group from EDHS 2016

Random effect model comparison	Model 0	Model 1	Model 2	Model 3
**Variance**	1.29	0.51	0.33	0.27
**Inter-cluster correlation(ICC)**	0.28	0.12	0.09	0.076
**Log likelihood ratio(LLR)**	− 1372	− 1405	− 3166	− 1301
**Deviance**	2744	2810	6332	2602
**Proportional change in variance(PCV)**	Ref	0.60	0.74	0.79
**Media odds ratio (MOR)**	2.90	1.90	1.70	1.60

At the community level, compared to the women in Tigray, women in Afar, Oromia, Somali, Benishangul, SNNPR, Harari, Addis Ababa, and Dire Dawa had 89.00, 61.00, 96.00, 58.00, 64.00, 85.00, 66.00, and 80.00% reduced intention to use modern contraceptive methods with AOR of 0.11(0.06–0.20), 0.39(0.23–0.64), 0.04(0.02–0.09), 0.42(0.25–0.72), 0.36(0.20–0.67), 0.15(0.08–0.28), 0.34(0.20–0.62), and 0.20(0.11–0.36) respectively; (Table 3).

Initially, the null model had 28.00% intention of contraceptive use appeared only due to the variation among clusters and the remaining left to the individual level. We sequentially developed models to handle such variations. And the inter-cluster variation was reduced by 1/4th to be only (7.00%). The PCV of intention to use contraceptive methods in the initial model was escalated to 2.90 times more variable due to the clustering effect. We described the modeling procedures to handle variation in Table 4. The variance, ICC, media odds ratio, and deviance decreased, while log-likelihood ratio and proportional change in variances increased illustration of fitness.

## Discussion

In this study, we analyzed the EDHS 2016 data to identify the intention of using contraceptive methods among reproductive-age women (15-49 years). We extracted a total of 5651 women from the dataset, among which the intent to use contraceptive methods was 44.11%. Compared to the other studies, intent to use contraceptive was 31.70% in Gahanna [8], 44.70% in Mozambique [28], 42.00% in Pakistan [29], 18.20% in Wellega-Ethiopia [29], 38.00% in Wolaita- Ethiopia [30], and 84.30% in Aksum Town-Ethiopia [30]. The discrepancies might be due to the comprehensive nature of our study and difference in setting, socio-demographic factors, and sampled areas (Table 1).

The mean age at first cohabitation in the data was 16.90 ± 3.99. In a study in Wolaita, it was 17.6 [16] and also reported in other studies < 16 years and ≤ 19 years [30, 31]. These might indicate its mean lies between 16 and 19 years. The mean number of children per participant was 4.50 ± 2.90. It was 4.90 ± 1.90 in Wolaita zone Ethiopia [30] and > 3.00 in North West Ethiopia [32] studies. 38.00% of women reported > 5.00 children per household in another study [9]. The mean looked similar throughout the country, and there were only a few differences among them. It might show the mean number of children per household is between these numbers, but difficult to conclude. Increasing family planning-related resources and making the intended women access the service might improve that. However, the mean ideal number of children reported by the women was 4.77 ± 2.00, which was coincided with the total ever-borne number of children. Conversely, it is inconsistent compared to the finding in Aksum town (2.00 ± 1.50) [32]. In evidence, 35.50% of women had five-plus ideal plans for the number of children [30]. With all efforts to increase family planning in the last 20 years in Ethiopia, many inconsistent reports demonstrate the need for more consistent interventions.

More than 2/3rd of the women were illiterates. The number was not shy relative to another study in the country, where it was 63.00% [9]. It was also 32.00% in Wolaita Sodo town [16], 43.20% in the Tigray region [18], and 62.10% in another study [16]. The discrepancies in the findings indicated education might be the main reason behind the dissimilarities in intention to contraception.

It was even expressive when only 2.40% of participants used newspapers/magazines for obtaining information. Learning less education deprives women of information access in developing countries. Uneducated women might not be aware as educated ones to use services available for reproductive health. Only 43.66% of the women had health facilities visit in the last 12 months, and only 27.68% of the women had formal work. Studies revealed that non-working women do not attend school and had less intention to use contraceptive methods [13, 16]. Good socioeconomic status factors were usually associated with health services utilization and birth planning, while the opposite might be true. In underdeveloped countries, women have no work, are poorly educated (both couples), and economically poor ( [5, 33–35]. These are the concerns that need to be managed as the main factors to solve utilization problems) (Table 1).

The significant clustering renowned in this study was another approach that explained, the percentage distribution of intention to use contraceptive methods in the country was not happened by chance. The intent to use contraceptives methods have clustered in Northwest Ethiopia [10] (Table 2).

It sparked some questions about whether the distribution followed some socio-demographic paths (Figs. 1, 2, 3, and 4). Evidence designated that the Northern part of the country is Orthodox believers, the Eastern part Muslims, and the southern and western parts were Protestants mostly [36, 37] (Table 2). The intention to use contraceptives was higher in 15-19 years than in all other age groups. This finding has support from another study in Ethiopia [30]. The possible explanation might be due to contraceptive use intention at an early age or before marriage. The current educational motivation due to different international efforts like the sustainable development goals might influence these things. Participants in the study in Gahanna showed the reverse intention compared to our study; the participants with primary education as their highest education had no intent to use contraceptive methods in that study [2]. That might indicate an intention to learn and use contraception was correlated; however, the status of the relationship was not clear. In other words, the finding is consistent with the result in North West Ethiopia. It means primary education influence might be different in different African countries. Women with a higher number of children showed more tendencies to use contraceptive methods, which is more global among many studies [9, 31]. Women who obtained information on contraceptive methods in health facilities became the other groups to show intention. It indicates that healthcare availability or accessibility increased counseling by health professionals, and that affected the intent to use contraceptive method [38] (Table 3).

Women with increased age at first cohabitation had better intention to use contraceptive methods compared to the younger. However, in some African countries, age at sexual debut has influenced future contraceptive use than the age at first cohabitation [39, 40]. The inconsistent information might be due to the difference in legality concept of sexual intercourse and cut year of marriage; where most women might report that only after marriage for a cultural reason. Compared to the Tigray region, less intention was observed almost in all others regions especially, in the Southwest and Eastern parts. It might signpost, the Tigray region has better contraceptive methods awareness and has good experience to learn from [29, 32, 41] (Table 1). Despite these findings, the current study has many limitations of the sampling distribution. We handled the clustering effect from different locations during analysis through pooled frequencies and multilevel analyses (Table 4). Although we managed all these, other limitations like removing data without coordinates, using third-party data, and reference of data back to 2016 might be some other limitations that need consideration while applying our findings.

## Conclusion

The intent to use contraceptive methods was distributed differently in different regions. Socioeconomic and demographic factors might be the reasons. Education and socio-cultural aspects need serious considerations to increase engagement in the services that include family planning. Tigray had good intentions and contraceptive uses; thus, using the region as the focal for experience sharing for others. Education showed a virtuous correlation with the intent to use contraception. So, educating women might increase the intention and use of contraceptive methods. Promoting women to start cohabitation at a later age could improve their intent to use contraceptive methods. From the result, we learned high intention among women who have started cohabitation later. Additionally, employing and empowering the women might improve the intention and contraception utilization since only less than 1/3rd of the women were currently working during the survey.

## Data Availability

The dataset supporting the conclusions of this article is available in the EDHS repository, (available from Measure DHS web site http://www.measuredhs.com).

## Abbreviations

WHO: World Health Organization
EDHS: Ethiopian Demographic Health survey
AOR: Adjusted odds ratio
CI: Confidence interval
SNNPR /SNNP: South Nations Nationalities people Region
EHNRI: Ethiopian Health Nutrition and Research Institute
NRERC: Review Board and the National Research Ethics Review Committee
